# A new look at an old virus: patterns of mutation accumulation in the human H1N1 influenza virus since 1918

**DOI:** 10.1186/1742-4682-9-42

**Published:** 2012-10-12

**Authors:** Robert W Carter, John C Sanford

**Affiliations:** 1 FMS Foundation, 877 Marshall Rd, Waterloo, NY, 13165, USA; 2 Horticulture Dept., NYSAES, Cornell University, Geneva, NY, 14456, USA

**Keywords:** Influenza, H1N1, Swine flu, Mutation accumulation, Pandemic, Evolution, Error catastrophe

## Abstract

**Background:**

The H1N1 influenza A virus has been circulating in the human population for over 95 years, first manifesting itself in the pandemic of 1917–1918. Initial mortality was extremely high, but dropped exponentially over time. Influenza viruses have high mutation rates, and H1N1 has undergone significant genetic changes since 1918. The exact nature of H1N1 mutation accumulation over time has not been fully explored.

**Methods:**

We have made a comprehensive historical analysis of mutational changes within H1N1 by examining over 4100 fully-sequenced H1N1 genomes. This has allowed us to examine the genetic changes arising within H1N1 from 1918 to the present.

**Results:**

We document multiple extinction events, including the previously known extinction of the human H1N1 lineage in the 1950s, and an apparent second extinction of the human H1N1 lineage in 2009. These extinctions appear to be due to a continuous accumulation of mutations. At the time of its disappearance in 2009, the human H1N1 lineage had accumulated over 1400 point mutations (more than 10% of the genome), including approximately 330 non-synonymous changes (7.4% of all codons). The accumulation of both point mutations and non-synonymous amino acid changes occurred at constant rates (μ = 14.4 and 2.4 new mutations/year, respectively), and mutations accumulated uniformly across the entire influenza genome. We observed a continuous erosion over time of codon-specificity in H1N1, including a shift away from host (human, swine, and bird [duck]) codon preference patterns.

**Conclusions:**

While there have been numerous adaptations within the H1N1 genome, most of the genetic changes we document here appear to be non-adaptive, and much of the change appears to be degenerative. We suggest H1N1 has been undergoing natural genetic attenuation, and that significant attenuation may even occur during a single pandemic. This process may play a role in natural pandemic cessation and has apparently contributed to the exponential decline in mortality rates over time, as seen in all major human influenza strains. These findings may be relevant to the development of strategies for managing influenza pandemics and strain evolution.

## Background

At the close of World War I, the H1N1 influenza A virus swept the world [1]. During the 1917–1918 pandemic, approximately 40% of the human population was infected, with a death rate above 2%. It is estimated that this virus killed more people than died in the world war that was just then ending. Mortality rates have dramatically declined since then [2], but the H1N1 flu has persisted. As a zoonotic pathogen, the influenza virus is able to infect multiple species. It is generally thought that aquatic waterfowl are a primary natural influenza reservoir [3], where there are usually no clinical symptoms [4], and where low level transmission probably perpetuates the viral pool [5]. All 14 influenza subtypes are maintained in waterfowl [5].

H1N1 has had an interesting history. Derivatives of the original virus circulated in humans and swine until 1957, when the human strain went extinct. In 1977, a version identical to those circulating in NE Europe in the early 1950s reappeared in Anshan, China and subsequently spread across the world [5-7]. In 2009, a swine H1N1 jumped to the human population, causing a widespread pandemic. This has increased concern that H1N1 might mutate into a more virulent form. However, since the pandemic of 1917, this has not happened. In fact, H1N1-related human mortality has declined very dramatically and very systematically [2]. Apart from the 1917 pandemic, H1N1 has failed to cause any severe global pandemic, and human H1N1 essentially went extinct from 1957–1977. Since its re-introduction, it has remained a relatively minor cause of influenza mortality [2]. This applies also to the 2009 outbreak, which caused relatively few deaths in those areas with good reporting systems in place [8].

It is therefore reasonable to ask if the striking reduction in H1N1 mortality might be due, in part, to natural attenuation resulting from deleterious mutation accumulation. Herd immunity is undoubtedly an important factor in reduced H1N1 mortality since 1918, but this may not be sufficient to explain the continuous decline in H1N1-related mortality over multiple human generations or the eventual extinction of the viral strain. Likewise, improved medical treatments, such as antibiotic treatment for flu-related pneumonia, were certainly a significant factor reducing H1N1 mortality, but these do not appear to fully explain the nature of the pattern of mortality decline seen for H1N1. For example, the exponential decline in mortality began before the invention of antibiotic treatment.

The literature suggests RNA viruses should be inherently subject to mutational degeneration [9-13]. This includes the bacteriophage MS2 [14], the tobacco etch virus [15], HIV [16-19], dengue virus type-2 [20], Ebola [21,22], and SARS [23,24]. Some have suggested that intentionally increasing the rate of mutation accumulation (“lethal mutagenesis”) may be a way to control viral epidemics by hastening strain extinction [25-30]. There is some long-term historical evidence that supports the concept of natural viral attenuation through mutation accumulation [2], and theoretical studies using numerical simulation strongly support the concept of natural and accelerated genetic attenuation of RNA viruses [13].

The influenza genome consists of eight RNA segments totaling over 13,100 nucleotides. These code for up to eleven distinct proteins, two with alternate reading frames and one through alternate splicing. Each of the eight RNA segments has its own history of reassortment, inheritance, and mutation [5]. In the same way, each of the major serotypes (e.g., H1N1, H2N2, H3N2) has its own rate of mutation and history of reassortment. Almost all of the influenza genome is protein coding, and 5.7% (749 nucleotides) of the genome codes for two protein products simultaneously (Table 1). Currently, there are thousands of fully-sequenced influenza genomes available in public databases, including a reconstructed version of a 1918 H1N1 genome. Most of these have collection dates and lineage information associated with them. Given this large body of data, it becomes feasible to test the attenuation model using mutation accumulation rates, non-synonymous amino acid changes, changing dN/dS ratios, changing transition/transversions ratios, and changes in codon specificity over time.

**Table 1:** Protein products of a consensus H1N1 influenza genome

**Segment**	Length^*^	**Protein**	**ORF**^+^	Amino Acids^*^
1	2280	PB2	1-2280	759
2	2274	PB1	1-2274	757
		PB1-F2	95-367	90
3	2151	PA	1-2151	716
4	1701	HA	1-1701	566
5	1497	NP	1-1497	498
6	1410	NA	1-1410	469
7	982	M1	1-759	252
		M2	1-27,716-982	97
8	838	NS1	1-693	230
		NS2	512-838	108

^*^Average full-length or predicted full-length product, ^+^relative to start codon (ATG).

Previous genetic studies examining the history of the influenza virus have performed extensive phylogenetic analyses of influenza genomes [8,31-35]. They have shown considerable nucleotide diversity among circulating strains, given clear evidence for adaptive selection of antigenic variants [36-42], and have shown that most of the major innovations within the flu genome have occurred via reassortment [5], by which one flu strain has recombined with another strain and obtained a segment of RNA from the second strain.

Influenza phylogenies are odd, in some respects, consisting of one main trunk with many short and short-lived side branches [36,39,42,43]. There are several factors that influence strain diversity, including the mutation rate, selection, drift, and cross-immunity-mediated competition between strains [39]. Feguson, *et al*. [39] felt that strain-transcending immunity was essential in restricting viral diversity. Ito, *et al.*[42] thought that the many side branches died out because they could not compete with the main-trunk lineage viruses. Others have noted the temporal extinction of circulating strains upon the introduction of new strains or serotypes [5]. Whatever the reason, a hierarchy of strain robustness is evident in the history of influenza viruses.

While phylogenetic studies can build robust family trees, they do so by focusing only on a limited number of “informative” genomic locations [44]. Even though the influenza genome is broken into eight separate RNAs, unless an individual is infected with two strains simultaneously (providing an opportunity for reassortment), all eight sections are inherited as a set in a form of linkage [36]. Thus, neutral and slightly deleterious mutations are carried along with those mutations under positive or purifying selection. This gives us enough information to make many phylogenetic inferences, and we have a wealth of data telling us the history of the various viral lines, but these phylogenetic techniques ignore genetic change within a larger portion of the genome in order to focus on the phylogenetically-informative sites.

There is abundant evidence for multiple substitutions in specific places that are, in turn, sites of active selection [42], but what about the rest of the genome? Fitch, *et al.*[36] noted that the main strategy of influenza viruses was to outrun the immune system of the host by maintaining a high mutation rate. They wondered “why such a virus does not accumulate so many deleterious mutations as to die of its own ineptitude.” They further wondered if the only reason influenza viruses continued to circulate in mammalian species was because of a sort of “gene therapy” through reassortment with stronger, less mutated avian viruses.

The present study is an attempt to elucidate more fully the nature of mutational change in this historically important human pathogen.

## Methods

Accession numbers for all available complete flu genomes were obtained from the Influenza Research Database [45] as of June 1, 2012. This list was then compared to those available at FluGenome.org [46]. To create a list of the 2009–2010 H1N1 outbreak versions, we used the collated genome list from Kedwaii, *et al.*[8]. Using these two accession lists, sequence data were obtained from GenBank [47]. We removed non-human/swine, non-H1N1, and incomplete genomes from this list, obtaining 3,755 human and 351 swine genomes. Because there are few indels in these flu genomes, a simple first-pass alignment algorithm, written in Perl with the BioPerl toolkit [48], was used. Essentially, we visually picked out a 6-10-nucleotide candidate region close to the beginning of each of the eight strands of the flu genome that appeared mostly conserved. The algorithm scanned each set of sequences for this segment and added an appropriate number of spaces to the beginning of each for a rough alignment. Using BioEdit [49], we simply looked for sequences that were misaligned and inserted gaps where necessary to complete the alignment, paying close attention to codon usage. In difficult and ambiguous regions, we chose to minimize the number of changes necessary to produce each variant, similar to the methodology of Carter [44].

Once an alignment for each of the eight sections of the flu genome was completed, we used another Perl program to scan through the alignment and compare each sequence to a reference strain. Point mutations were counted individually while indels, irrespective of size, were counted as a single change [44]. There are several different ways to calculate sequence differences (discussed in [50]), but this simple measure is sufficient for the purposes of this paper. Due to variations in technique among the various studies that produced these sequences, the ends of the alignment tended to be ragged. For this reason, we compared only the section of complete alignment between each sequence and the reference genome. The few internal reassortments [32] that have occurred in H1N1 were easily seen in the alignments, but these also made up a small fraction of all sites. We did our best to create a consistent alignment through these regions.

We began by analyzing mutation accumulation during the human H1N1 outbreak of 2009–2010, using strain California/04/2009 as a reference. This was the first sequenced genome of the outbreak included in Kedwaii, *et al.*[8]. We then calculated the extent of mutational divergence of all subsequent genomes over the duration of the outbreak.

We did a similar, but much larger analysis of mutation accumulation in all human H1N1 strains from 1918 to 2012, using the 1918 “Brevig Mission” reference genome (A/Brevig Mission/1/1918) as a reference. This sequence was missing the last 480 nucleotides of segment 4. We amended this by appending the ending sequence of another 1918 H1N1 virus, A/South Carolina/1/18(H1N1). These two strains had only one nucleotide difference in the ORF of segment 4 (C/T at position 238). Thus, they are essentially identical and we do not expect to have introduced extra mutations in this way.

Using the amended 1918 Brevig Mission virus as a reference and including all human and porcine viruses in the database, we calculated SNPs, indels, transitions, transversions, non-synonymous amino acid changes, dN/dS ratios, predicted protein lengths (for all 11 proteins), the normalized codon scores (NCS) and relative synonymous codon usage (RSCU) [51] score for each predicted protein of each genome. To calculate the normalized codon score, we downloaded the frequency of each of the 64 codons in the human, duck and domestic pig genomes from the Codon Usage Database [52]. We then created a predicted amino acid chain from each of the respective open reading frames. We calculated NCS for each protein by simply summing the frequency of each codon, as it is used in each of the three species, and then dividing by the total number of codons in the proteins produced. RSCU is simply the number of times any particular codon appears, divided by the number of times synonymous codons appear in that genome. Anhlan, *et al*. [51] were the first to apply RCSU to influenza genomic data, but they only studied one of the eleven protein products of the H1N1 genome and did not analyze changes over time. Here, we extend their techniques to create a time series in which one can better study shifts in codon usage due to mutation accumulation.

## Results

**Figure 1:**
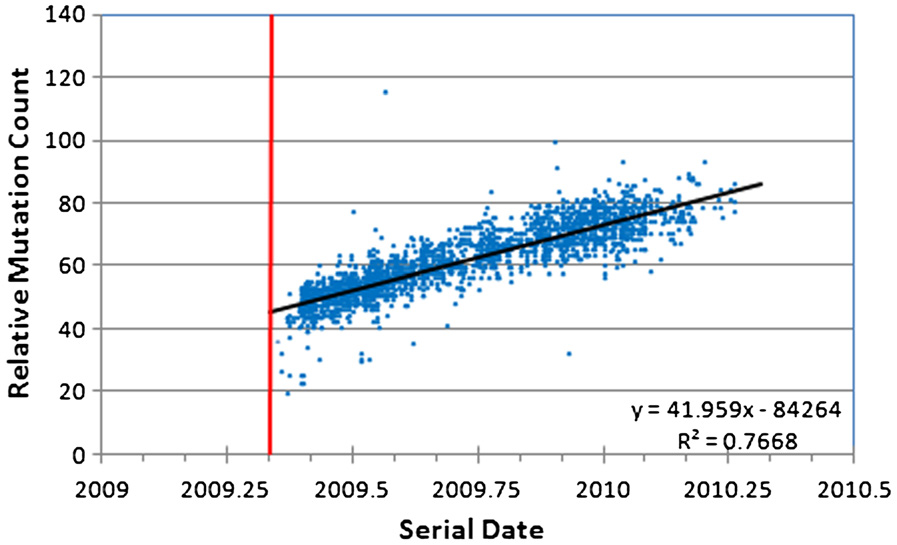
**Mutation accumulation within the 2009 A**/**H1N1 influenza strain during the course of the 2009**/**2010 flu season.** The earliest sequenced genotype (California/04/2009) was used as the baseline for comparison with all subsequent genotypes (mutation counts reflect divergence from that starting sequence). The vertical red line indicates the date the reference sequence was collected. This sequence is not included because it, by definition, has a mutation count of zero and ignores contemporaneous sequence variation.

During the 2009–2010 H1N1 outbreak, mutations within H1N1 accumulated at a relatively constant rate (Figure 1). The strains sequenced at the end of the pandemic had approximately 80 more mutations than the 2009 reference genotype. The line of best fit reveals a slope of 42.1 mutations accumulated per year (3.2 × 10^-3^ mutations per site per year). Given that the data were collected from viral strains circulating worldwide, the correlation between mutation count and the date of collection was surprisingly good (r^2^ = 0.77).

**Figure 2:**
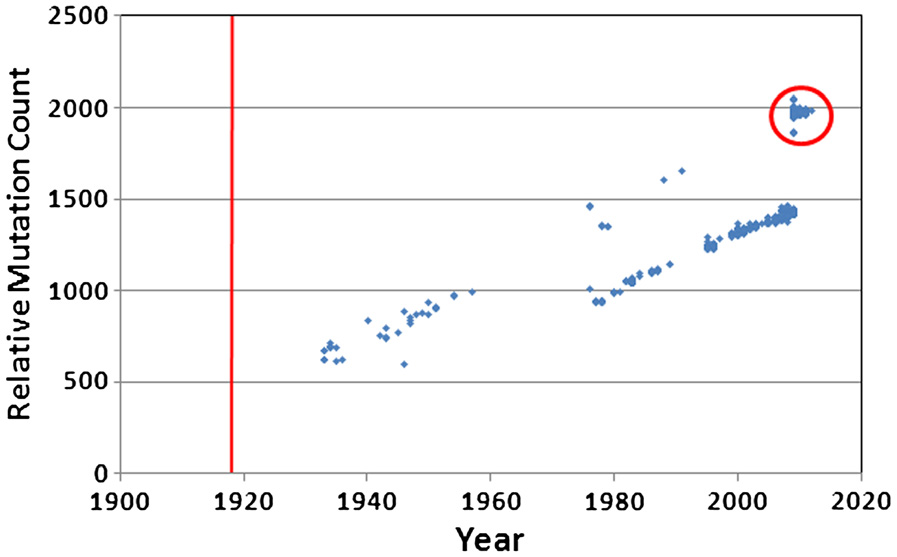
**Mutation accumulation in H1N1 in deeper time.** The published Brevig Mission strain from 1918 was used as the baseline for comparison with all available human H1N1 genomes. There are two distinct trend lines in the data. The 2009–2010 outbreak samples and additional samples from 2011–2012 are circled.

The linear accumulation of mutations during the 2009 outbreak clearly also extends to the longer-term accumulation of mutations during the entire history of H1N1 (Figure 2). During the last century there has been a remarkably constant increase in mutation count within the H1N1 virus population, except for a striking discontinuity between 1957 and 1976. This discontinuity reflects the extinction of the human H1N1 strain in the mid 1950s, followed by the re-introduction of the strain in 1976, presumably from a researcher’s freezer. Even though most reports (e.g., [53]) indicate the re-introduction year was 1977, our data suggests the year of re-introduction was 1976. This is based upon the isolate A/New Jersey/1976, which very clearly falls in line with the rest of the re-introduced lineage. Notice that after re-introduction in 1976, the human H1N1 mutation count and the rate of accumulation resumed exactly where it left off just before the extinction occurred in the 1950s.

Our data clearly show that some non-frozen H1N1 genotypes occasionally appeared in the human population after H1N1 dropped from public concern after 1957 (Figure 2). Nine H1N1 strains that do not belong to the “frozen” lineage arose in the human population between 1976 and the 2009 H1N1 outbreak (A/Wisconsin/301/1976, A/New Jersey/8/1976, A/California/10/1978, A/California/45/1978, A/Albany/8/1979, A/Memphis/1/1979, A/USSR/46/1979, A/Ohio/3559/1988, and A/Maryland/12/1991). These fall on the same mutation-count trajectory as the H1N1 circulating before the 1957 extinction. However, these nine strains appear to represent repeated transmission events from pigs to humans that failed to cause any pandemic (the porcine lineage had no extinction event, and hence no pause in mutation accumulation). Nearest neighbor calculations (data not shown) indicate these strains are not a continuation of the human lineage. They cluster tightly with the 2009–2010 outbreak porcine viruses, all of which are more closely related to the 1918 isolate than they are to the human lineage viruses. Thus, we included the 2009–2010 viruses and the nine isolated, non-outbreak viruses in the “porcine” category. In this same figure, we can see that genotypes from the 2009 outbreak and after (Figure 2, circle) fall directly on the trajectory of the non-frozen lineage. This clearly shows that the 2009 genotype was not derived from the 1976 re-introduction virus. Kedwaii, *et al.*[8] affirmed earlier conclusions that the 2009 genotype was due to a reassortment between two swine viruses, an H1N2 and an H1N1, from different continents, but this did not affect the mutation accumulation curve. Regardless, general attenuation due to genome-wide mutation accumulation might best explain the very low mortality [54-56] associated with the 2009 pandemic. The earliest 2009 outbreak strain had already accumulated 1,889 mutations compared to the 1918 strain.

The most obvious place to test how reassortment might affect the mutation accumulation curve is with the 2009–2010 “swine flu” outbreak samples. Despite the fact that the viral line has been attributed to reassortment with swine viruses, with partial contribution from earlier reassortments between swine and avian strains [8], it does not display a jump in mutation count. Reassortment can produce novel antigenic variants, but it does not reverse the majority of mutations, for they have accumulated in the non-reassorted areas of the genome.

**Figure 3:**
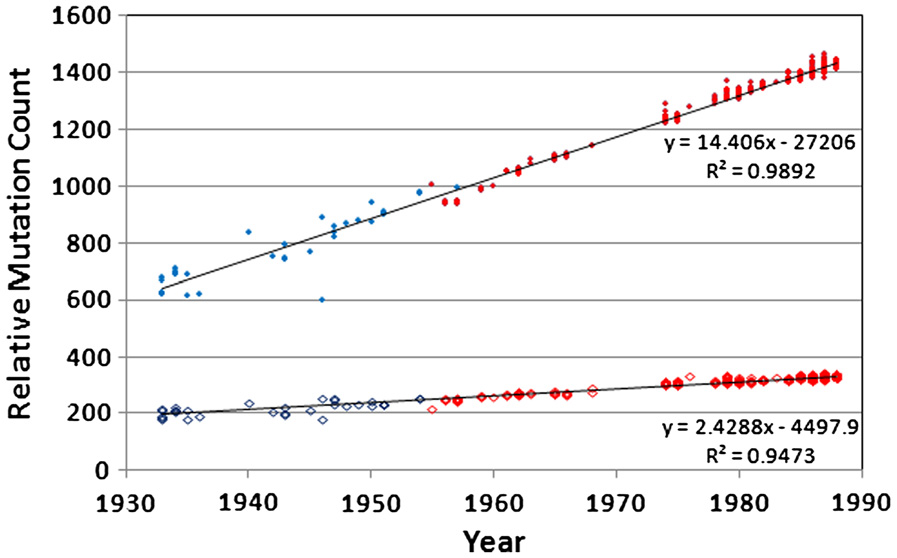
**Mutation accumulation in human H1N1**, **using the 1918 Brevig Mission strain as a reference.** Sequences derived from the 1976-reintroduction (red symbols) were moved back in time by 21 years to adjust for an apparent period of dormancy, resulting in highly-linear mutation accumulation curves. Filled symbols = nucleotide changes. Hollow symbols = non-synonymous amino acid changes.

After we identified the direct 1918 human lineage viruses, we were able to adjust the dates of the later samples to account for a period of dormancy (Figure 3). When we shifted the “frozen” samples back 21 years (i.e., sequences from 1976 were assigned a recalibrated date of 1955), we see the data lines up almost perfectly (r^2^ = 0.989; mutation rate = 14.4). The exact amount of time-frame adjustment was not critical; adjustments ranging from 20 to 30 years all gave excellent alignments, with correlation coefficients above 98%. The observed accumulation rate of roughly 14 mutations per year (1.1 × 10^-3^ mutations per site per year) is more or less consistent with previous estimates (e.g., [39,57]). The accumulation of non-synonymous mutations in human H1N1 genomes is also shown in Figure 3. The non-synonymous mutations are not as abundant, as expected, but they are still accumulating at a rapid and constant rate. There were an average of 2.4 amino acid changes per year (0.6 × 10^-3^ mutations per codon per year).

Our data show that the lineage re-introduced in 1976 was the only significant human H1N1 strain from 1976 until 2009. The mutation count trajectory of the H1N1 lineage from the 1976 re-introduction stops abruptly in 2009 and does not reappear in any of the 353 H1N1 genomes published from mid-2009 through mid-2012. Human H1N1, the remnant of the 1918 lineage, appears to have gone extinct a second time.

The data in Figure 3 can be used to estimate the time of arrival into the human population of the first H1N1 virus. Extrapolating total mutation count linearly backwards to zero yields a year of first introduction into the human population of approximately 1893. Interestingly, this would be in time to explain the 1889–1890 “Russian” flu outbreak, which has been considered by some to possibly be the first H1N1 outbreak (no sequence or serotype data are available for that event). While our data strongly suggest that the ancestral genotype for all human and porcine H1N1 influenza strains (and by extension, all human influenza strains), entered the human population as early as the 1890s, others have estimated the arrival date to be one or more decades later [5]. Our data strongly confirm that all human H1N1 viruses derive from a single, very recent common ancestor, just as others have concluded prior to this study [1]. Given an estimated arrival date of 1893, we infer that, by 1918, H1N1 would have accumulated roughly 375 (25 years × 15 mutations/year) mutations since the initial genotype invaded the human population. This depends, of course, on the exact arrival date.

Within the entire human H1N1 lineage, only 56% of all nucleotide sites were invariant, indicating intense mutation pressure. Before its apparent extinction in 2009, the human H1N1 lineage had accumulated approximately 1400 mutations (mostly point mutations), including 320 non-synonymous mutations, compared to the 1918 genotype. Across all 3,755 H1N1 genomes isolated from humans (i.e., including the outbreak and non-outbreak porcine versions), we found that only 41.8% of all nucleotide sites were invariant. Multiple variant forms (i.e., 3-fold and 4-fold degeneracy, meaning 3 and sometimes 4 different nucleotides observed at a given site) occurred at 21% of the variable loci, further suggesting that the mutations were primarily non-adaptive. When we included the porcine H1N1 genotypes, only 36.1% of sites remained invariant, with a similar distribution of nucleotide degeneracy. Because the re-introduced H1N1 lineage had accumulated fewer mutations than the porcine H1N1 strains, the disappearance of the human lineage and the subsequent dominance of the 2009 porcine strain in the human population resulted in even more divergence from the 1918 stain: over 1900 nucleotide differences (approximately 15% divergence) and 325 amino acids differences (approximately 7% divergence in protein sequence).

The approximately 90-nucleotide PB1-F2 protein is coded on the second genomic segment and overlaps completely the PB1 coding region [58,59] Table 1. PB1-F2 has been implicated in increasing host death rate. Yet, not all influenza viruses code for this protein, so it is not intrinsically necessary for the cycle of infection. In the case of H1N1, the majority (98.2%) of genomes in this study are predicted to fail to produce a full-length version of the protein, including most of the human versions after 1948 and none of the porcine versions prior to 1987. For this reason, only 10 of the 11 influenza proteins were included in RSCU and NCS calculations. All genomes were predicted to produce fully-formed PB2, PB1, PA, HA, NP, NA, M1, M2, and NS1 proteins. Only 14 genomes were expected to fail to produce a functioning NS2 protein due to mutation in the start codon, but all of these had another potential in-frame ATG only a few codons upstream and/or downstream. For this reason, we assumed these viruses would also produce an NS2 protein of proper length.

**Figure 4:**
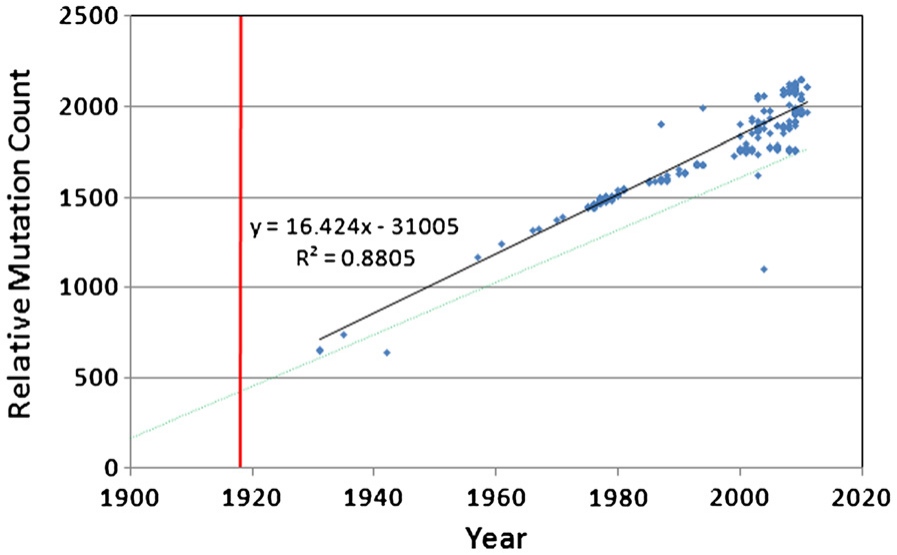
**Mutation accumulation in porcine H1N1.** Solid line = porcine regression. Dashed line = the human regression line from Figure 3. There is considerably more variability among the porcine versions of H1N1, and there was a significant difference in the two slopes (ANCOVA, p=0.0001).

Mutation accumulation in swine H1N1 since 1918 is shown in Figure 4, with the human regression line (with “frozen” linage adjusted for 21 years of dormancy) for comparison. Regression analysis (ANCOVA) indicated a significant difference in the slope (p = 0.0001) of the lines of best fit for the viruses obtained from the two species. Although it is clear that the swine versions have more variability towards the end of the sampling period, they also have a higher mutation rate in general, even after adjusting the human data for the 21 year period of dormancy.

We note that many mutations appeared and disappeared several times over the life of H1N1. The data are too sparse over most of the range to know if this is due to repeat mutations at common loci or incomplete genetic sampling of the circulating H1N1 viruses. However, over the 2009–2010 H1N1 outbreak, Kedwaii, *et al*. [8] demonstrated near-complete lineage loss among the circulating viruses during a single season. Bedford, *et al*. [33] had similar results in their study of influenza A (H3N2). It was noted earlier that the phylogeny appears as a main trunk with many short-lived side branches. If most mutations are lost from the population quickly, variations that appear several times over the course of H1N1 are most likely due to mutations occurring multiple times in the same place. We do not have enough sequence data to get a full picture of strain diversity in most years, but this will be interesting to pursue as more data become available.

Even though each genomic segment has a slightly different mutation/substitution rate, strong, genome-wide patterns are evident, including a declining ratio of non-synonymous to synonymous codon changes (dN/dS), a declining ratio of transition to transversion mutations (Ti/Tv), and even changes in codon bias. Additional file 1 includes these data on a segment-by-segment basis.

**Figure 5:**
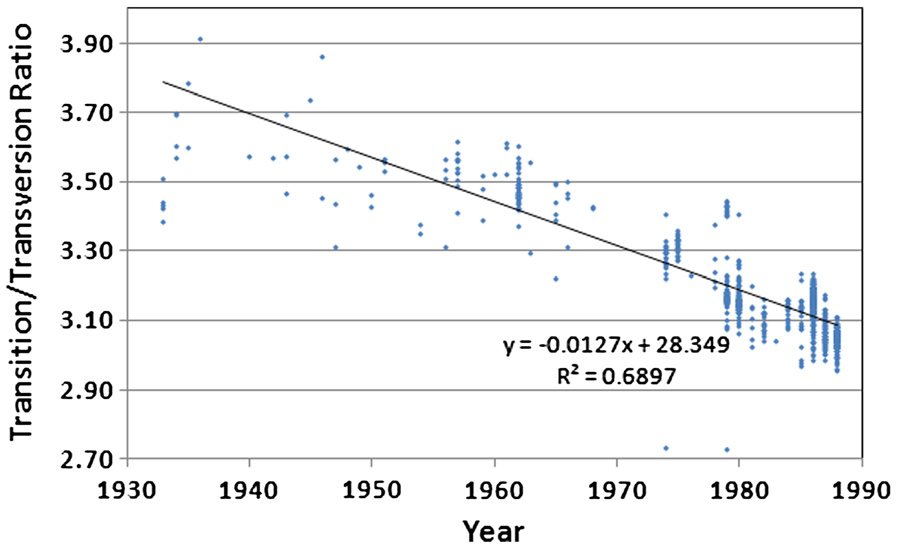
**Changes in the transition**/**transversion ratio over time for the direct 1918 human H1N1 lineage viruses,after adjusting the** “**frozen**” **portion of the lineage (1976**–**2009)for a 21**-**year dormancy period**.

Figure 5 illustrates a continuous decline in the Ti/Tv ratio during the entire history of the human H1N1 lineage. This suggests there may be increasing selection against transversions over time, perhaps reflecting some degree of truncation selection as the genome becomes more attenuated and as extinction approaches. A transversion is 2.7 times more likely to cause a non-synonymous change than a transition, and is also more likely to change RNA 3-dimensional configuration. Since we do not see a change in the rate of accumulation of amino acid substitutions over time (Figure 3), we suspect that the heightened selection against transversions may be more strongly associated with RNA architectural constraints.

Over the history of the human H1N1 lineage, the accumulation of non-synonymous mutations was highly linear, after correcting the “frozen” samples for 21 years of dormancy (Figure 3). While it is true that this simple distance metric applied to amino acid changes would be expected to underestimate evolutionary relationships at large distances due to an increasing likelihood of multiple changes at individual loci, applying the standard Poisson or Gamma corrections [60] made no improvement to the fit of the regression to two significant figures. We therefore conclude that the relationship is indeed linear, at least over the sampled timescale.

**Figure 6:**
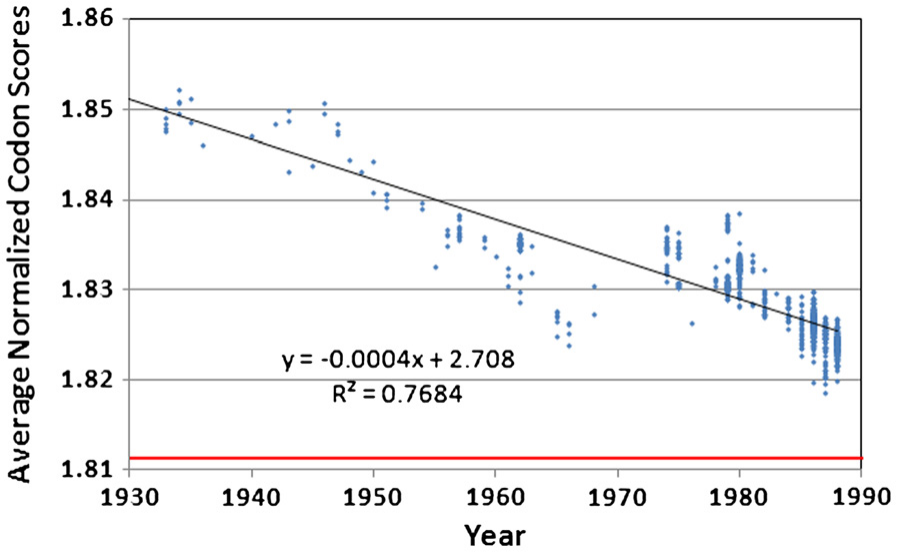
**Average normalized codon scores for the direct 1918 human H1N1 lineage viruses**, **after adjusting the “frozen”portion of the lineage (1976**–**2009)backwards in time by 21 years.** The red line at 1.812 indicates the score that would be obtained from a series of proteins with the same amino acid content as those produced by H1N1, but with randomized codons.

Analysis of the normalized codon scores (NCS) clearly shows that codon preference degenerated continuously throughout the entire history of the human H1N1 line (Figure 6). All viruses in our database are more duck-like in their codon usage and most (99.8%) are more human-like than pig-like, but codon preference moved toward randomized codon use and away from the codon preference of the human, swine and duck hosts. Thus, the virus is not adapting to use the codon preference of any of the host genomic environments, even when residing in a single species for 70 years (the duration of the human H1N1 lineage, after correcting for 21 years of dormancy) with no evidence for the introduction of new genetic material through reassortment. These data are further evidence that the genome-wide changes are not adaptive, and are in fact degenerative. Non-synonymous changes had little effect on the NCS scores. The 345 non-synonymous amino acid changes between 1918 and 2009 (A/Pensacola/INS235/2009) had a negligible effect (to three significant figures) on the final result for this sequence.

**Figure 7:**
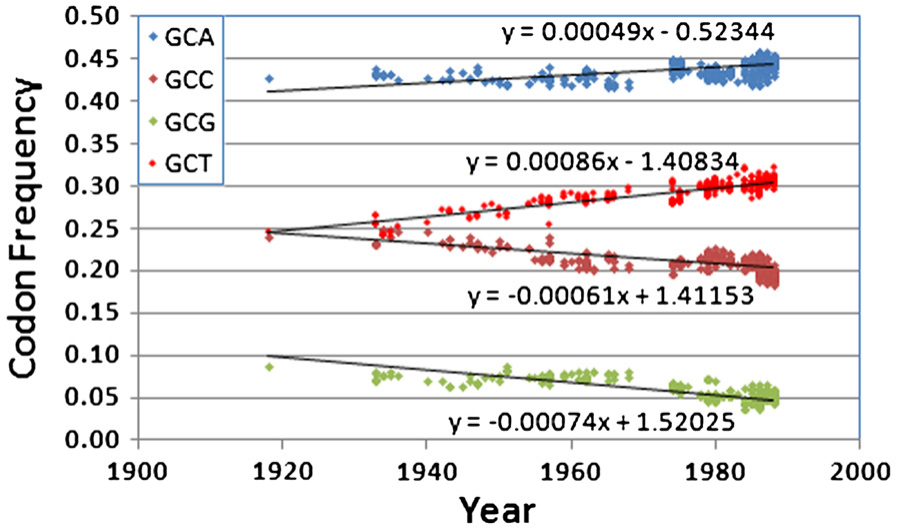
**Relative synonymous codon usage in the direct 1918 human H1N1 lineage for the four alanine codons,after adjusting the “frozen”portion of the lineage (1976**–**2009)by 21 years.** Note that the two decreasing in frequency over time are exactly matched by the two increasing over time (i.e., non-synonymous changes are having a negligible effect). Also, three of these lines (GCA, GCC, and GCG) indicate these codons are drifting even farther away from the human average.

Relative synonymous codon usage (RCSU) patterns support the attenuation hypothesis as well (Figure 7, Additional file 2). Most codons changed in frequency in a highly consistent manner over the life of the 1918 human H1N1 strain. Those with the greatest rate of positive change over time were CAA (glutamine), ATC (isoleucine), and GTA (valine), and two stop codons, TAA and TAG. Each of these was balanced by a strong decrease in a synonymous codon: TGA (stop), ATT (isoleucine), CAG (glutamine), and GTG (valine).These changes are due to simple transition mutations, possibly associated with some mutational bias. The most stable codons all encoded either serine (TCA, TCC, TCT), or threonine (ACT, ACC, ACA, ACG). More than half of all codons (33 out of 62) were changing in frequency in a direction away from the average human codon usage, including three of the four in Figure 7. All but one codon (ACC) had a regression line significantly different from a slope of zero at the p<0.05 level, and most (56 out of 62) scored p<0.0001. Thus, both RCSU and NCS reveal a clear and systematic erosion of codon bias over time.

## Discussion

It has generally been assumed that any non-neutral mutations within the influenza genome have arisen as selective adaptations and generally help drive influenza toward a stronger and more dangerous pathogen (in terms of either pathenogenicity or transmissibility) [41]. This was probably the basis for the extreme caution exhibited during the 2009–2010 H1N1 outbreak. There is a general perception that, given enough time, H1N1 might mutate into a stronger pathogen, and hence might create another catastrophic pandemic, as it did in 1918.

Selection is evident in H1N1 and other influenza genomes. In certain sites of the HA1 genomic segment of H3, non-synonymous substitutions occur at more than twice the rate as in other sites [39]. This is seen as a major signature of adaptive change, but deleterious mutations in the areas not under selection are carried along with the ones under positive selection [36]. In this light, some perceive H1N1 to be a growing threat, with a new outbreak being just a matter of time. Despite this common perception, a more lethal version of H1N1 has not arisen via mutation within the human population during the last 90+ years. This is significant. The two major human influenza pandemics since 1918 did not arise due to mutations within H1N1, but arose via horizontal transmission of new genetic material from bird influenza strains, creating recombinant viruses. They were also less lethal than the 1918 version. It is true that the population had a degree of residual immunity and was not as immunologically naïve as it was in 1917–18, but selection has still not been able to generate a devastating pandemic from the remnants of that which swept the world at the close of WWI.

In this paper, we examine an alternative point of view regarding mutation accumulation within H1N1. We suggest that, while specific adaptive mutations commonly occur within the H1N1 virus, many more deleterious mutations are accumulating than beneficial mutations, even when there is strong selection. Consequently, H1N1 appears to have been in very gradual error catastrophe throughout its history.

Our results strongly confirm the widely recognized fact that all past and present human and swine H1N1 influenza strains derive from the 1918 strain. By extension, this applies to other human influenza strains, including H3N2 and H2N2 [1].

Human influenza has such a high mutation rate that, even within a single host individual, the virus quickly becomes a genetically diverse “swarm” [61,62]. Yet, globally human H1N1 influenza is monophyletic and all current variation is of recent origin. This is only possible if almost all human influenza lineages rapidly go extinct. Moreover, we present strong evidence that the H1N1 genome has been systematically degenerating since 1918. This is evidenced by continuous, systematic, and rapid changes in the H1N1 genome throughout its history. For example, there was an especially rapid and monotonic accumulation of mutations during a single pandemic (Figure 1). Similarly, there was a continuous and rapid accumulation of mutations over the entire history of the virus (Figures 2 and 3), including a similar steady increase in non-synonymous amino acid substitutions (Figure 3). While mutations accumulated in the human H1N1s, there was a parallel accumulation of mutations in the porcine H1N1 lineage (Figure 4). Fitch, *et al.*[36] also showed a linear mutation accumulation curve. The “gnarled trunk” of Ito, *et al.*[42] should be buried in our data as well, but it will be swamped by the pervasive, genome-wide accumulation of mutations not under active selection.

Within the human lineage, there was a systematic decline in the Ti/Tv ratio (Figure 5). In addition, there was a very consistent loss of codon specificity over time (Figures 6 and 7). We show compelling new evidence supporting the extinction of human H1N1 in the 1950s, its subsequent re-introduction in 1976, and an apparent second extinction event of the human H1N1 lineage in 2009. Strain extinction has often occurred when new strains appeared, including H1N1 replacing the circulating H3-like strains in 1917, H2N2 replacing H1N1 in 1957, and H3N2 replacing H2N2 in 1968 [5]. To our knowledge, we are the first to document the replacement of the re-introduced human H1N1 with reassorted swine H1N1 in 2009. All this is consistent with the genetic attenuation hypothesis, and we feel this is the most fundamental explanation for the very smooth, systematic, and exponential decline in H1N1 mortality rates since 1918 [2].

### Relevance and potential objections

In light of these findings, what are the medical implications? Does mutation accumulation really have anything to do with virulence? Simonsen, *et al*. [2] showed mortality statistics for three influenza strains over multiple years (H1N1 from 1918 to 1987; H2N2 from 1958 to 1962; and H3N2 from 1968 to 1995). Even though there is some debate concerning the mortality burden imposed by influenza viruses [63], there has clearly been a continuous exponential decline in influenza-related mortality over time, and this is true for all three major serotypes. Since there is a strong linear correlation between mutation count and time (Figures 2 and 3), and since there is also a close correlation between declining virus-related death rates and time, there is obviously also a correlation between mutation count and reduced death rates. Reduction in mortality may be due to many other factors, including herd immunity, advances in medicine, and advances in hygiene, but would these other factors be expected to follow so tightly the time courses seen in Simonsen?

There have been major medical advances since 1918, and these have clearly been a factor in reducing H1N1-related mortalities. Therefore, the correlation between mutation count and reduced H1N1 mortality might be considered spurious by some. However, while it is certainly true that medical intervention has greatly improved in the developed world since 1918, such medical intervention has been much more limited in the rest of the world. Second, the observed decline in mortality is a remarkably smooth curve, while medical advances have occurred in bursts (e.g., the breakthrough in antibiotics, and the more recent development of antivirals). Third, each of the great influenza pandemics (1918, 1956, 1968) involved the emergence of a new viral strain, which then followed its own exponential decline in mortality but within its own timeframe. This uncouples reduction in mortality and stage of medical advance. Finally, the correlation between the exponential decline of H1N1-related mortalities and the linear increase in H1N1 mutations is only one of our evidences for the genetic attenuation of H1N1. Our other evidences include: a) the extinction of all human influenza strains existing prior to the H1N1 strain; b) the apparent extinction of the human lineage of H1N1 in 1956, and then again apparently in 2009; and c) the erosion of H1N1 codon specificity, approaching random codon usage.

It is our contention that all human influenza strains undergo natural attenuation due to mutation accumulation. It is too early to tell if the remaining versions of the 2009–2010 outbreak viruses will do the same, but it is likely given the known history of change in the various influenza genomes.

The decline in codon bias is especially significant for several reasons. First, since the frequency of codon usage is positively correlated with tRNA availability in the cell [64], the increased use of rare codons is expected to negatively affect protein translation rates. Alternatively, Li, *et al*. [65] did not notice any decrease in translational efficiency based on codon choice in bacteria, but they did see effects caused by mutation towards other genomic control motifs (i.e., anti-Shine Dalgarno sequences). Part of codon bias deals with a cell’s avoidance of controlling factors that do not directly deal with translation rates (e.g., CG dinucleotides). Thus, there are multiple ways a disruption of codon bias might negatively impact the functionality of a particular stretch of any nucleic acid. Second, even though the codon usage in ducks and humans is similar [51], although less so for swine [52], this might affect the ability of a virus to cross species lines (after cellular antigenic recognition is taken into account). Third, Anhlan, *et al*. [51] entertained several hypotheses, including that the virus was avian in origin but transferred to pigs before it jumped to humans. Our data clearly indicate that all H1N1s studied are more duck-like in their codon usage, although we cannot comment about a pre-human swine intermediate based on our data. Finally, since we see an obvious decay in codon bias over time when compared to codon usage in either human, duck, or pig, it is clear that H1N1 is not evolving toward optimal codon usage in any of these species but is slowing drifting away from optimal translational efficiency. We concur with Anhlan, *et al*. that, “the issue of codon usage seems to be much more important at least for influenza viruses than previously thought.”

During the last 100 years, the H1N1 influenza genome has diverged from the original genotype by roughly 15%. Might the approximately 1,900 nucleotide substitutions be primarily attributed to the genetic drift of perfectly neutral variations? This seems unlikely for several reasons. First, a viral genome of approximately 13,000 nucleotides does not have room for very much neutral RNA. Not only did 15% of the genome change, but polymorphisms arose across more than 50% of the genome. This strongly points to extreme mutational pressure, high enough, reasonably, to threaten error catastrophe. Second, if some significant portions of the viral genome are neutral, deletions of such portions of the viral genome should be regularly seen, and selection should favor such deletions, rapidly producing smaller genomes. There is no evidence of significantly smaller influenza genomes. Indeed, there is little evidence of deletion in any of the 2009–2010 genomes compared to the 1918 version. The only major indels occurred among the oldest samples (prior to 1948) in the sixth genomic segment (neuraminidase, or NA), but all of these represented deletions compared to the 1918 genome and all later genomes. Third, it is now known that even synonymous mutations are not always neutral. Even though they may not directly affect protein sequence, they can affect RNA stability, RNA architecture, speed of translation, and protein folding. Fourth, there should only be a finite number of nucleotide positions that are perfectly neutral. Because of this, neutral divergence should quickly approach a limit, causing the rate of divergence from the original genotype to slow, but this is not seen. Finally, the extensive genetic changes observed simply do not appear to be phenotypically neutral; they are tightly correlated with rapid fitness decline, attenuation, extinction of most circulating strains, and even more frequent sub-lineage extinction events.

Might the observed divergence be primarily due to adaptive mutations? We feel that the 15% divergence must be primarily non-adaptive because adaptation should occur rapidly and then reach a natural optimum. Yet, we see that divergence increases in a remarkably linear manner. Furthermore, the virus does not seem to be converging on a new optimal genotype since polymorphism remains extreme (over 50%), since many polymorphic sites have more than two alleles, and since codon specificity is declining over time. Codon patterns can inform us about the origin of the virus, and they tell us that H1N1 is not only drifting away from that original codon use, but it is also drifting away from the host codon preferences. When grown in mouse cell culture, 2009 H1N1 viruses exhibited variation in replication rates, virulence, and pathogenicity, but these did not match the severity of clinical symptoms in humans [66]. Thus, at this time, the exact relationship between mutation load and the severity of infection remains unknown. Yet, selective adaptation is limited to only those amino acids that produce significant phenotypic effects [42], and, since a viral genome in the absence of reassortment is essentially a single linkage block, it is expected that many more than just adaptive mutations occur. Some of the changes might be due to selection, but the majority certainly are not.

Is it feasible that natural selection might fail to remove a large number of deleterious mutations? It is well known that numerous factors can cause a breakdown in the selective removal of deleterious mutations. These factors include a high mutation rate, a high rate of random loss, limited sexual recombination, genetic bottlenecking, and mutations with very small fitness effects. All of these factors should be especially pronounced in an RNA virus such as influenza and all of these are either previously known or documented here. The genetic changes in H1N1 appear non-directional and are distributed quite uniformly across the genome (Additional file 3), consistent with an accumulation of low-impact deleterious mutations. Error catastrophe and lethal mutagenesis are already recognized as a threat to any RNA virus. These facts, combined with the dramatic decline in H1N1 mortality and the very high rate of H1N1 strain extinction, all very strongly indicate that most of the genetic divergence from the original H1N1 genotype has been due to fixation of slightly deleterious mutations.

Could H1N1 ever back-mutate into a strain such as the ancestral genotype that caused the catastrophic 1917–1918 pandemic? Given that the modern strains of H1N1 have diverged from the original 1918 strain by nearly 2000 mutations, that many of these mutations should be slightly deleterious [67], and that natural selection was unable to stop their continuous accumulation in the first place, it is difficult to imagine how mutation/selection might ever restore full virulence. Reassortment might bring in new material, but thus far this has only applied to a limited section of the genome, and reassortment today occurs in a very different mutational/genomic context than that of 95 years ago. It is often thought that a high mutation rate translates to rapid adaptation and evolution, yet the reverse seems more commonly true. Deleterious mutations often interfere with selection for the more rare beneficial mutations [67,68]. Thus, the rapid accumulation of mutations in all H1N1 lineages should logically lead to their eventual extinction.

The origin of human H1N1 influenza is unknown, but it is generally reasoned that it invaded the human population from a natural reservoir [5,51], most likely an aquatic waterfowl, with pigs as a possible intermediate host. In light of the strong tendency toward natural genetic attenuation which we document here, we suggest that the natural reservoir most likely involves a very quiescent viral state, as might occur within a host where there is very little viral replication, and hence much lower mutation rates. It would be very interesting to know the rate of influenza mutation accumulation in waterfowl.

Can reassortment explain these findings? Based on our mutation count analysis, there is no evidence for reasortment in the human H1N1 lineage, and it has gone extinct, apparently twice. From other studies, various influenza strains are obviously derived from reassortment, but all this does is set the mutation clock back a little. Any reassortment between a “fresh” virus and a high-mutation-count virus will inevitably lead to, at best, an averaging of the mutational load of the two. The 2009–2010 “swine flu” virus shows evidence of multiple reassortment events in a limited portion of its genome, and it was more robust than the lingering human H1N1 strain that it replaced, but it carries a great number of non-adaptive and presumably deleterious mutations. Reassortment between two viruses of different immunological character might preserve the less degraded genome, but only temporarily.

Might pandemics be shortened by artificially accelerating the rate of genetic attenuation? The continuous and linear accumulation of mutations within a single influenza lineage, as was seen in H1N1 during the 2009–2010 influenza season (in which about 0.3% of all nucleotides mutated), supports the concept that natural genetic attenuation may be an important factor in the natural cessation of influenza pandemics. Thus, the possibility of an artificial acceleration of mutation rate deserves further investigation, and may suggest new avenues of research in terms of pandemic management [13]. It is clear that natural selection is strongly at work in the influenza genome. This can be seen by preservation of all the basic proteins and functions of the virus, in spite of the fact that every possible point mutation happens in every human individual during the course of an infection. A large fraction of all deleterious mutations clearly must be selected away. Likewise, the emergence of major antigenic variants shows that positive selection is operational. It is also clear that genetic drift is strongly in operation, with a major viral bottleneck happening at each transmission from one human host to the next, and perhaps at the start of each local outbreak [33], ensuring that most unique genotypes are very quickly lost. Yet, in addition to selection and drift, it also appears there is very strong mutational pressure on the influenza genome, potentially leading to lethal mutagenesis in most strains, and a gradual, natural genetic attenuation of human influenza in general.

Read and Taubenberger [69] called the origin of human H1N1 an “enigma” whose riddle was not yet solved. Like them, we see this as an unsolved riddle and we can only hope that our data might bring us one step closer to understanding the origins of this important disease.

## Conclusions

Sequence analysis of historical and modern genomes of influenza H1N1 reveal a great deal about the history of the virus. The most recent common ancestor existed only about 120 years ago, and there has been universal extinction of all earlier human influenza strains. The rate of mutational divergence from that original genotype has been very constant both in human and porcine H1N1 strains (roughly 14–16 mutations fixed per year). Modern H1N1 strains have diverged from the original genotype by roughly 1,900 fixations (more than 15% of the genome has mutated). Roughly 7% of the amino acids have mutated. Mutation accumulation is also associated with the historical exponential decline in H1N1 human mortalities, which may suggest significant genetic attenuation, as might arise due to very slow error catastrophe.

Sequence analysis confirms that the human H1N1 strain went extinct in 1957, but was reintroduced in 1976, apparently from a specimen frozen in the early 1950s. The resulting “frozen” lineage appeared less mutated compared to all other contemporary H1N1 strains (i.e., porcine strains and a few rare H1N1 strains appearing in humans but not derived from the frozen strain). Consistent with the genetic attenuation model, the frozen human H1N1 lineage disappeared in 2009, and may now be extinct.

It appears that the H1N1 strains currently in circulation are significantly attenuated, and cannot reasonably be expected to back-mutate into a non-attenuated strain. The greatest influenza threat, therefore, is the introduction of a non-attenuated strain from some natural reservoir. This suggests that a better understanding of the origin of such non-attenuated strains should be a priority [5]. Our findings suggest that new strategies that accelerate natural genetic attenuation of RNA viruses may prove useful for managing future pandemics and, perhaps in the long run, precluding the genesis of new influenza strains.

## Abbreviations

NSCU: Non-synonymous Codon Usage; NCS: Normalized Codon Scores.

## Competing interests

The authors declare that they have no competing interests.

## Authors’ contributions

RC wrote the computer programs and performed most of the analyses. JS was responsible for the development of the initial idea and interpretation of the data generated. Both authors contributed equally to the preparation of the manuscript. All authors read and approved the final manuscript.

## Supplementary Material

Additional file 1 H1N1 strain variation data using the 1918 Brevig Mission virus as a reference, in .csv format. Click here for file

Additional file 2 Relative Synonymous Codon Usage (RSCU) data, in .csv format. Click here for file

Additional file 3 H1N1 genomic variation data, in .csv format. Click here for file
